# Supplementary material for: SyNDI: synchronous network data integration framework
Source: BMC Bioinformatics. 2018 Nov 6;19:403. doi: 10.1186/s12859-018-2426-5 (PMC6219086; doi:10.1186/s12859-018-2426-5)
Supplement: Supplementary file 5 — Files of the example presented in Additional file 4. This additional file is a zip package that contains all files used in Additional file 4. Detailed descriptions of each of these files are presented in a README file included in this zip package. (GZ 30411 kb) [file 12859_2018_2426_MOESM5_ESM.gz › SyNDITools/Meme2Fimo/meme_4.9.0_4.tar.gz!/meme_4.9.0/doc/examples/fimo_example_output_files.tgz!/fimo_example_output_files/fimo.html]

FIMO Results


---

|  |  |  |
| --- | --- | --- |
| **Database and Motifs** | **High-scoring Motif Occurences** | **Debugging Information** |

  
  

---

**FIMO - Motif search tool**


---

FIMO version 4.8.1,
(Release date: 7 PDT 20)

For further information on how to interpret these results
or to get a copy of the FIMO software please access
http://meme.nbcr.net

If you use FIMO in your research, please cite the following paper:  
Charles E. Grant, Timothy L. Bailey, and William Stafford Noble,
"FIMO: Scanning for occurrences of a given motif",
*Bioinformatics*, **27**(7):1017-1018, 2011.

---

**DATABASE AND MOTIFS**


---

DATABASE
../../../tests/common/crp0.fasta  
Database contains
18
sequences,
1890
residues

MOTIFS
../../../tests/common/crp0.meme.html
(nucleotide)

| MOTIF | WIDTH | BEST POSSIBLE MATCH |
| --- | --- | --- |
| +1 | 18 | AATTGTGACGTAGATCAC |
| -1 | 18 | GTGATCTACGTCACAATT |
| +2 | 8 | CGGTGGGG |
| -2 | 8 | CCCCACCG |
| +3 | 11 | CCTGGTGTGCC |
| -3 | 11 | GGCACACCAGG |

Random model letter frequencies
(from non-redundant database):
  
A 0.275 C 0.225 G 0.225 T 0.275

---

**SECTION I: HIGH-SCORING MOTIF OCCURENCES**


---

- There were
  24
  motif occurrences with a
  p-value less than
  0.0001.
- The p-value of a motif occurrence is defined as the
  probability of a random sequence of the same length as the motif
  matching that position of the sequence with as good or better a score.
- The score for the match of a position in a sequence to a motif
  is computed by summing the appropriate entries from each column of
  the position-dependent scoring matrix that represents the motif.
- The q-value of a motif occurrence is defined as the
  false discovery rate if the occurrence is accepted as significant.
- The table is sorted by increasing p-value.

| Motif | Sequence Name | Strand | Start | End | p-value | q-value | Matched Sequence |
| --- | --- | --- | --- | --- | --- | --- | --- |
| 3 | lac | − | 33 | 43 | 2.27e-07 | 0.000776 | `CCTGGGGTGCC` |
| 1 | ara | − | 59 | 76 | 4.92e-07 | 0.000591 | `AAGTGTGACGCCGTGCAA` |
| 1 | lac | + | 9 | 26 | 4.92e-07 | 0.000591 | `TAATGTGAGTTAGCTCAC` |
| 3 | trn9cat | + | 36 | 46 | 5.03e-07 | 0.00086 | `CCTGGTGTCCC` |
| 1 | malt | + | 41 | 58 | 5.86e-07 | 0.000591 | `AATTGTGACACAGTGCAA` |
| 1 | ilv | − | 43 | 60 | 1.61e-06 | 0.00106 | `AATTGAGGGGTTGATCAC` |
| 1 | deop2 | + | 60 | 77 | 1.74e-06 | 0.00106 | `AATTGTGATGTGTATCGA` |
| 1 | pbr322 | − | 57 | 74 | 2.98e-06 | 0.00151 | `ATCTGTGCGGTATTTCAC` |
| 1 | uxu1 | + | 17 | 34 | 4.31e-06 | 0.00186 | `TGTTGTGATGTGGTTAAC` |
| 1 | ce1cg | − | 65 | 82 | 4.97e-06 | 0.00188 | `TTTTGTGAAAACGATCAA` |
| 1 | trn9cat | + | 84 | 101 | 6.13e-06 | 0.00206 | `AAATGAGACGTTGATCGG` |
| 2 | ilv | + | 5 | 12 | 6.67e-06 | 0.0235 | `CGGCGGGG` |
| 1 | gale | − | 46 | 63 | 8.61e-06 | 0.00261 | `AAGTGTGACATGGAATAA` |
| 1 | tnaa | − | 75 | 92 | 1.05e-05 | 0.00275 | `AAATGTGAATCGAATCAC` |
| 1 | crp | − | 67 | 84 | 1.12e-05 | 0.00275 | `TAATGTGACGTCCTTTGC` |
| 1 | cya | + | 50 | 67 | 1.27e-05 | 0.00275 | `AGGTGTTAAATTGATCAC` |
| 1 | male | + | 14 | 31 | 1.27e-05 | 0.00275 | `TTCTGTAACAGAGATCAC` |
| 2 | male | + | 41 | 48 | 1.48e-05 | 0.0261 | `CGGTGGGG` |
| 1 | ompa | + | 48 | 65 | 1.54e-05 | 0.00311 | `ATGCCTGACGGAGTTCAC` |
| 1 | malk | + | 61 | 78 | 2.09e-05 | 0.00396 | `TTTCGTGATGTTGCTTGC` |
| 1 | tdc | − | 82 | 99 | 2.22e-05 | 0.00396 | `ATATGTGCGACCACTCAC` |
| 1 | tnaa | + | 71 | 88 | 2.5e-05 | 0.00421 | `GATTGTGATTCGATTCAC` |
| 1 | tdc | + | 78 | 95 | 5.8e-05 | 0.00925 | `ATTTGTGAGTGGTCGCAC` |
| 1 | bglr1 | + | 76 | 93 | 8.35e-05 | 0.0126 | `AACTGTGAGCATGGTCAT` |

---

**DEBUGGING INFORMATION**


---

Command line:

```
/Users/cegrant/meme/bin/fimo --oc fimo_example_output_files ../../../tests/common/crp0.meme.html ../../../tests/common/crp0.fasta
```

Settings:

```
|  |  |  |
| --- | --- | --- |
| output directory = fimo_example_output_files | MEME file name = ../../../tests/common/crp0.meme.html | sequence file name = ../../../tests/common/crp0.fasta |
| allow clobber = true | compute q-values = true | output threshold set = false |
| text only = false | scan both strands = true | max sequence length = 250000000 |
| output threshold = 0.0001 | threshold type = p-value | pseudocount = 0.1 |
| verbosity = 2 |  |  |
```

This information can be useful in the event you wish to report a
problem with the FIMO software.

---

**Go to top**
